# Fault slip and identification of the second fault plane in the Varzeghan earthquake doublet

**DOI:** 10.1007/s10950-018-9734-0

**Published:** 2018-02-22

**Authors:** Samar Amini, Roland Roberts, Mohammad Raeesi, Zaher Hossein Shomali, Bjorn Lund, Zoya Zarifi

**Affiliations:** 1 Department of Earth Sciences, Uppsala University, Villavagen 16, 75236 Uppsala, Sweden; 2 SeisAnalysis AS, Bergen, Norway; 3 Department of Earth Sciences, University of Western Ontario, London, ON Canada

**Keywords:** Earthquake source estimation, Teleseismic body waves, Slip inversion, Coulomb stress changes, Asia

## Abstract

An intraplate earthquake doublet, with 11-min delay between the events, devastated the city of Varzeghan in northwestern Iran on August 11, 2012. The first *Mw* 6.5 strike-slip earthquake, which occurred after more than 200 years of low seismicity, was followed by an *Mw* 6.4 oblique thrust event at an epicentral separation of about 6 km. While the first event can be associated with a distinct surface rupture, the absence of a surface fault trace and no clear aftershock signature makes it challenging to identify the fault plane of the second event. We use teleseismic body wave inversion to deduce the slip distribution in the first event. Using both P and SH waves stabilize the inversion and we further constrain the result with the surface rupture extent and the aftershock distribution. The obtained slip pattern shows two distinct slip patches with dissimilar slip directions where aftershocks avoid high-slip areas. Using the estimated slip for the first event, we calculate the induced Coulomb stress change on the nodal planes of the second event and find a preference for higher Coulomb stress on the N-S nodal plane. Assuming a simple slip model for the second event, we estimate the combined Coulomb stress changes from the two events on the focal planes of the largest aftershocks. We find that 90% of the aftershocks show increased Coulomb stress on one of their nodal planes when the N-S plane of the second event is assumed to be the correct fault plane.

**Electronic supplementary material:**

The online version of this article (10.1007/s10950-018-9734-0) contains supplementary material, which is available to authorized users.

## Introduction

On 11 August 2012, the city of Varzeghan in northwestern Iran was shaken by two earthquakes of magnitudes *Mw* 6.5, at 12:23 GMT, and *Mw* 6.4, 11 min later. Three hundred fatalities and more than 3000 injuries were officially reported, and 30,000 people were made homeless. Although the earthquakes were not of great magnitude, they were felt over a large area in northwestern Iran, Azarbaijan, and Armenia.

Correctly identifying the location and orientation of the two faults is important for understanding the mechanisms controlling these events, potentially with significant relevance for hazard assessment in this area. The earthquake source area had no reported seismic history (Berberian and Yeats 1999), nor a known trace of a recently active fault. Field observations after the doublet reported an E-W trending fault trace (Copley et al. 2013), but these authors could not with certainty state which of the two events produced the surface rupture. However, Donner et al. (2015) argued that the surface rupture was produced by only the first mainshock since its fault plane is favorably oriented along the surface rupture trace.

In order to estimate the kinematic source parameters of the doublet, Masominia et al. (2014) applied a generalized inversion on local acceleration data assuming the Brune (1970) source model. The estimated source radii were 13.6 and 8.2 km with rupture duration times of 18.2 and 11 s for the first and second mainshocks, respectively. Akbarzadeh and Mahood (2015) and Mahood et al. (2014) used a stochastic finite fault method and found fault dimensions of about 10 × 15 km for the first mainshock. Using the combination of field mapping, remote-sensing, and optical image correlation, Copley et al. (2013) estimated the faulting in the Varzeghan doublet and suggested a spatially distributed deformation characterized by strike-slip faulting and a component of shortening. They also jointly inverted P and SH waveforms at teleseismic distances to find the focal parameters of the first mainshock. The strike, dip, and rake value they found is in close agreement with the global-CMT (http://www.globalcmt.org) solution, but the centroid depth and scalar seismic moment are far from that. In another study, Donner et al. (2015) estimated seismic moment tensors of the two mainshocks and the following large aftershocks (*Mw* ≥ 4.3) by inverting their surface wave data. Combining the regional seismic moment tensor with geomorphological analyses of the region, they suggest a temporally and spatially complex style of deformation. Very complex kinematic and tectonic deformation was also suggested by Ghods et al. (2015), who applied a combined methodology of structural geology, geomorphologic, and seismological approaches to construct a model of active faulting in the area. The slip distribution of the two mainshocks was also estimated by Yadav et al. (2016). They used coseismic offsets from GPS measurements at six permanent sites within epicentral distances of 25 to 70 km. Naturally, they could estimate only the cumulative offsets and the obtained slip model was simple and devoid of detailed features, possibly due to the limited number of GPS stations.

In this study, we use the teleseismic body wave inversion method of Kikuchi et al. (1993) to deduce the slip distribution of the first mainshock. The method has previously been applied to events such as the Tokachi-oki earthquake (Yamanaka and Kikuchi 2003) and a triple earthquake along Kurile subduction zone (Raeesi and Atakan 2009). The 11-min time delay between the two Varzeghan events, which have epicenters of 5 to 10 km apart (according to the various studies referred to above), suggests possible involvement of static stress triggering rather than dynamic mechanisms. Therefore, following studies on progressive faulting, e.g., the North Anatolian Fault (Stein et al. 1997) and the southern San Andreas fault (Lin and Stein 2004), we estimate Coulomb stress changes induced by the first event on the fault plane of the second event in order to elucidate the interaction between the events (Harris 1998). We also analyze the combined effect of the two mainshocks on the largest aftershocks following the hypothesis of Coulomb stress triggering in the production of aftershocks (Toda et al. 2011a).

## Seismotectonic setting

**Fig. 1:**
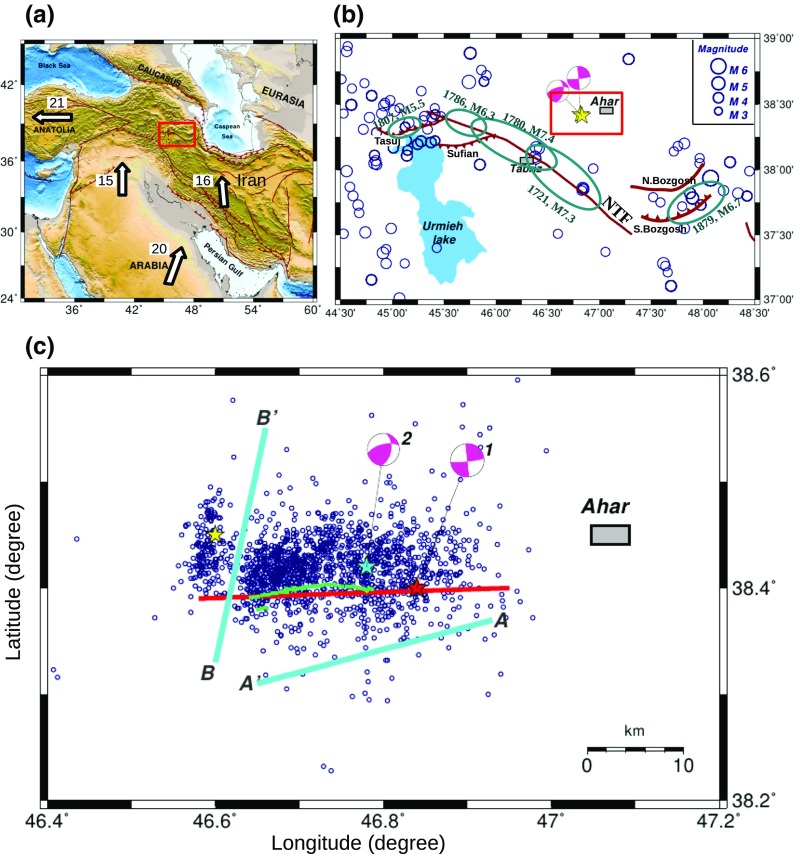
**a** Tectonic map of the Arabia-Eurasia collision. Arrows and the numbers indicate GPS-derived plate velocities (mm/year) relative to Eurasia (Reilinger et al. 2006). Red rectangle: the area shown in **b**. **b** Seismicity of northwestern Iran for magnitudes larger than three. Stars and the associated beach balls display the Ahar-Varzeghan double-earthquake. Circles: seismicity of the area from 1960 up to August 11, 2012, taken from ISC and IRSC (Iranian Seismological Center). Recent historical earthquakes and their meizoseismal areas are marked with green ellipses (from Berberian and Yeats 1999). Major faults in dark red. Gray rectangles are main cities. NTF North Tabriz Fault. Red rectangle: The area shown in **c**. **c** Aftershock activity for 3 months after the doublet according to the IRSC catalog for magnitude larger than 3. Red star and red line: epicenter and fault plane of the first event. Blue star and blue lines: epicenter and nodal planes of the second event. Location of epicenters are according to Ghods et al. (2015). Yellow star: location of the largest aftershock, *Mw* 5.6, that happened on November 7, 2012. Green lines mark the observed surface rupture trace reported by Faridi and Sartibi (2012)

Northwestern Iran is part of the Arabia-Eurasia collision zone where the northward convergence of Arabia toward Eurasia has caused complex tectonics and various structural features in a relatively small area. The rate of large-scale, north-south convergence is ~ 15 mm/year. on the southern margin of the Turkish-Iranian plate (at approximately 30° N, 40° E; Fig. 1a; Reilinger et al. 2006). The Arabia-Eurasia convergence is strongly oblique, see Fig. 1a, relative to the plate boundary, and the resulting deformation is mostly distributed between shortening on thrust faults and seismic slip on large strike-slip faults (Jackson 1992).

The North Tabriz Fault, which is the most important tectonic feature of NW-Iran, is a large strike-slip fault trending NW-SE along several right-stepping en-echelon segments with an overall length of more than 200 km (Karakhanian et al. 2004). The right-lateral motion on different segments of this fault absorbs about 8 mm/year of the convergence in the Arabia-Eurasia collision (e.g., Nilforoushan et al. 2003; Masson et al. 2007; Karimzadeh et al. 2013). Even though there is known historical activity (Berberian and Yeats 1999), recent earthquake data from 1960 to 10 August 2012 taken from the EHB (http://www.isc.ac.uk) and IRSC (Iranian Seismological Center, http://irsc.ut.ac.ir/) bulletins do not show any earthquake larger than magnitude 5 in NW-Iran, and the most recent major movement of the NTF was a sequence in 1721–1786 (Fig. 1b).

Despite the significant number of historical earthquakes on the NTF and the repeated destruction of Tabriz city, there are no known reports of previous activity in the Ahar-Varzeghan region where the double earthquake occurred (Fig. 1b). One possibility is that the apparent absence of seismicity in this area is due to incomplete historical information and lack of a dense seismic network. Geological maps of the area do include indications of a fault in the area of the doublet, suggesting that there might have been some historical events in the Ahar area, but circumstances such as the low population of the city of Ahar, that not all documents have survived or only limited damage was caused, could mean that events in historical time are absent in the existing documents.

## The double earthquake of August 11, 2012

The horizontal separation reported by the US Geological Survey (http://www.usgs.gov/) between the two main events was 10 km. Ghods et al. (2015) relocated the events and suggest a 6-km epicentral separation. We use Ghods et al. (2015) relocations in our analysis.

Table 1 lists the fault plane solutions of the double-earthquakes according to the global CMT solution (Dziewonski et al. 1981; Ekström et al. 2012). The first event (fault 1) had almost purely strike-slip, and the second event (fault 2) had oblique thrust motion. The nodal planes are distinguished by “a” and “b” in Fig. 1c. There were numerous aftershocks with magnitudes up to 5.6, lasting for almost a year after the mainshocks. According to the IRSC catalog, the aftershock activity covers an area of about 40 km × 30 km (see Fig. 1c).

**Table 1:** Fault plane solutions for the double-earthquakes according to the global CMT solutions. The hypocenter location is according to USGS

	(a) Strike/dip/rake (°)	(b) Strike/dip/rake (°)	Hypocenter depth (km)	Hypocenter latitude (°)	Hypocenter longitude (°)	Origin time (GMT)	CMT depth (km)	*Mw*	Scalar moment (N m)
Event 1	84/84/170	175/81/6	11.0	38.33	46.83	12:23′:17″	15.0	6.5	6.04e+18
Event 2	255/63/134	10/50/36	12.0	38.39	46.74	12:34′:35″	19.0	6.4	4.24e+18

“a” and “b” are the alternative nodal planes for the mainshocks

A presumed coseismic, E-W trending right-lateral strike-slip surface rupture with a small thrust component has been traced for ~ 12 km (Fig. 1c; Faridi and Sartibi 2012). The maximum observed surface deformation is estimated to be 70 cm right-lateral motion and 25 cm vertical with an average net slip of 72 cm (Donner et al. 2015). It is not completely clear which of the two events generated the surface rupture; however, it is favorably oriented along one of the nodal planes of the first event (as reported by global CMT), and it is difficult to associate it with the focal mechanism of the second event (see Fig. 1c). Following the argument in Donner et al. (2015), we agree that the surface rupture was most probably generated by the first event, and we will use this observation to constrain the first event’s slip model. The rupture plane for the first event, deduced from our slip inversion, is shown by the red line in Fig. 1c. For the second event, we aim to assess which of the nodal planes (marked as AA′ and BB′ on Fig. 1c) is the correct slip plane. For the second event, the nodal plane solutions are from the global-CMT, which are also in close agreement with the results of the deviatoric moment tensor inversion of Donner et al. (2015). Despite the interference of the coda of the first even with the signals from the second event, the global-CMT reports rather similar uncertainty estimates for these two mainshocks.

## Methodology

### Wave-form inversion

In order to estimate the slip distribution associated with the first mainshock, we used the body-wave waveform inversion code developed by Kikuchi et al. (1993). In this method, the fault plane is parameterized along the fault with pre-determined and constant azimuth and dip angles. The observed far-field displacement *u*_*j*_(*t*) at station *j* due to shear dislocation on a fault surface *S* can be represented as follows (e.g., Olson and Apsen 1982):

1 $$ {u}_j(t)={\sum}_{q=1}^2\underset{s}{\int }{G}_{qj}\left(t,\xi \right)\ast {\dot{D}}_q\left(t,\xi \right) d\xi, $$

where * means convolution in time domain, *G*_*qj*_ is the Green’s function for source at *ξ* and receiver at station *j*. $\dot{D_q}$ is the *q-th* component of spatiotemporal slip rate which can be written as a linear combination of space and time basis functions (Olson and Apsen 1982; Yagi and Fukahata 2011):

2 $$ \dot{D_q}\left(t,\xi \right)\simeq \sum \limits_{k=1}^K\sum \limits_{l=1}^L{a}_{qkl}{X}_k\left(\xi \right){T}_l\left(t-{t}_k\right), $$

Note that *q* specifies whether the slip component is along strike or dip. The coefficients *a*_*qkl*_ are to be determined. *X*_*k*_(*ξ*) is the space basis function at grid node *k. t*_*k *_in time basis function *T*_*l*_(*t* − *t*_*k*_) shows the time that rupture starts at grid node *k*. By inserting $\dot{D_q}\left(t,\xi \right)$ from Eq. (2) in Eq. (1), we obtain

3 $$ {u}_j(t)=\sum \limits_{q=1}^2\sum \limits_{k=1}^K\sum \limits_{l=1}^L{a}_{qkl}{T}_l\left(t-{t}_k\right)\ast \underset{s}{\int }{X}_k\left(\xi \right){G}_{qj}\left(t,\xi \right) d\xi \kern0.5em , $$

By using the following substitution,

4 $$ {H}_{qklj}={T}_l\left(t-{t}_k\right)\ast \underset{s}{\int }{X}_k\left(\xi \right){G}_{qj}\left(t,\xi \right) d\xi $$

we can rewrite Eq. (3) as

5 $$ {u}_j(t)=\sum \limits_{q=1}^2\sum \limits_{k=1}^K\sum \limits_{l=1}^L{H}_{qkl j}{a}_{qkl}\kern0.5em , $$

and in vector form as

6 $$ \mathbf{u}=\mathbf{Ha} $$

A non-negative least squares method is used to solve for the array of coefficients *a*_*qkl*_ subject to the constraint that the solution vector has non-negative elements, i.e., slip is zero or positive. A smoothness constraint is applied as prior information and a specific model among the group of models is objectively selected using the Akaike Bayesian information criterion (ABIC; Sclove 1987).

In general, the method finds the total slip at each node by linear summation of slip in both strike and dip directions of the fault plane, at different times. It uses a constant rupture velocity and assumes a simple predefined fault geometry which may differ from the real case, especially for major earthquakes.

### Coulomb stress changes

When slip occurs, it changes the stresses around the fault. To find the induced stress changes, we used the Coulomb 3 software (Toda et al. 2011b). This program calculates stress changes from fault displacements and is extensively used to evaluate the “Coulomb failure criterion” for points around the fault. This criterion describes the change in Coulomb failure stress (ΔCFS) on a receiver fault as the sum of changes in the shear stress (Δτ) and the normal stress (Δσ) scaled by the effective coefficient of friction (μ’);

7 $$ \Delta CFS=\Delta \tau -{\mu}^{\hbox{'}}\Delta \sigma $$

The Coulomb failure stress allows us to identify areas and fault orientations where the potential for future slip/failure has increased (ΔCFS > 0) or decreased (ΔCFS < 0) due to the slip in the main event. Results from this type of analysis have some limitations. For example, Eq. (7) does not consider transient pore pressure changes during slip; our knowledge of fault slip is not exact, so our estimates of ΔCFS contain uncertainties; and the criterion tells us only if the receiver fault has moved closer to failure, not how close to failure it is (which depends also on pre-existing stress). However, the method gives valuable insights into the mechanical consequences of earthquake slip.

The calculated stress changes were relatively insensitive to μ′ values between 0.4 and 0.8. Presented results use 0.6, appropriate for the upper crust (Byerlee 1978). For the regional stress directions, we used the results of fault kinematic inversion in the Ahar-Varzeghan area calculated by Ghods et al. (2015).

## Data

Waveform data from IRIS (Incorporated Research Institution for Seismology; http://www.iris.edu/) obtained from more than 100 stations at teleseismic distances (30°–70°), were used for the slip inversion. After correcting for instrument responses, a zero-phase band-pass filter from 0.015 to 0.5 Hz was applied to capture the body-waves. Then, poor-quality seismograms, identified by visual inspection and comparison of signals between stations, were rejected. After careful data selection, 84 P-waveforms and 63 SH-waveforms (at a total of 93 stations) remained for the inversion.

The CRUST2 model (Bassin et al. 2000) was used for the crustal structure at the receivers. For the source area, we have tested both regional and local velocity models (e.g., the local velocity model of Moradi et al. (2011)). Here, we present the results obtained with the regional model (listed in Table 2) which is routinely used by IIEES to locate events in Iran. This is also the velocity model used for relocation of the mainshocks and the aftershocks (Ghods et al. 2015). Our inversion experiments show that both velocity models result in very similar slip distributions. The initial fault plane solution for the inversion was taken from the global-CMT solution (Table 1).

**Table 2:** The 1D velocity model introduced by the International Institute of Earthquake Engineering and Seismology (http://iiees.ac.ir). The Vp/Vs ratio is 1.73

Top (km)	Vp (km/s)	Vs (km/s)
0	5.4	3.1
6	6.0	3.5
14	6.3	3.6
18	6.5	3.75
48	8.1	4.6

## Slip results

We conducted separate and combined inversions of P and SH data. Separate time windows of 25 s starting 5 s before the onset of the direct P (on vertical components) and SH phases (on tangential components) at each station were chosen for the inversion. The size of the fault plane (first mainshock) was fixed to 32 km along strike and 20 km along dip from the free surface, in accordance with the approximate horizontal extent and depth distribution of the aftershocks and the size of the fault estimated from GPS measurements (Yadav et al. 2016). The fault plane was discretized using 4 km nodal spacing. Larger grid spacing limits possible spatial resolution of slip and the resolving power of the data is not sufficient for smaller grid spacing. This yields eight nodes along strike and five nodes along the dip direction. The source time function at each grid point was described by a triangle of 1 s half duration where its amplitude was determined by the inversion. The strike and dip of the fault is fixed during each inversion, while the rake angle at each node is allowed to vary in the range of ± 45°. Our choices of strike and dip were also investigated by inverting with different input parameters (see below). The depth of the mainshock was controlled manually, to provide a good fit to the seismic data, during repeated inversions. In the inversions, least squares misfit calculated as Σ(*x*_obs_ − *x*_cal_)^2^/Σ(*x*_obs_)^2^, where *x*_obs_ and *x*_cal_ are observed and model data, respectively, was minimized.

**Fig. 2:**
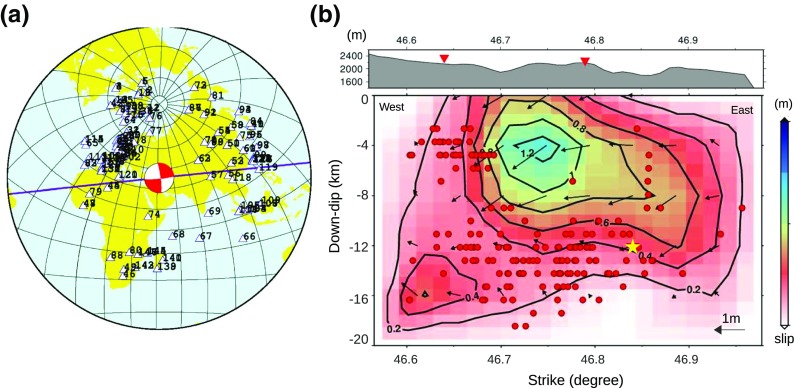
**a** Station distribution used for joint P-SH slip inversion of the first event. **b** The estimated slip pattern of the first mainshock using both P and SH data. Horizontal axis is distance along the strike and vertical axis is distance along the dip of the fault planes. Yellow star marks the hypocenter location. Color pattern and contour lines show slip values; arrows are slip vectors. Red circles are the first 3 months of aftershocks with magnitude larger than 3 (from Ghods et al. 2015). The topography profile along the strike of the fault is plotted above the slip map, and the red triangles on it marks two ends of the surface rupture exposure

Data is available from a considerable number of stations. However, the station distribution is much denser to the north, especially the northwest, than to the south (Fig. 2a). To reduce the risk of bias due to the non-uniform station distribution, we assessed a station-weighting scheme where we assigned higher weights to stations in areas with sparse distribution and lower weights to stations in dense areas. However, the resulting slip models do not change noticeably with different weighting arrangements (see online resource 1, Fig. S1) and we continued the analyses with uniform weights for all the stations.

In general, for strike slip faults, the SH amplitudes are larger than the P amplitudes so it is appropriate to consider applying some weighting scheme to the different phases when inverting SH and P data together. One possibility is to normalize the SH and P waveforms according to, e.g., their amplitude values or residual misfits. However, different assumptions regarding signal and noise characteristics imply different choices of what type and level of weighting to use, implying a risk that our choice of weighting may affect the reliability of the inversion process negatively, for example if the P wave data is less reliable (more noisy) than the SH. We experimented with weights determined by the relative amplitude ratio of P and SH waves at each station, and also based on the maximum absolute amplitudes of each phase. However, because the final slip maps were significantly affected by the choice of weights we decided to avoid any arbitrary choice of weighting and assigned equal weight to all the waveforms.

**Fig. 3:**
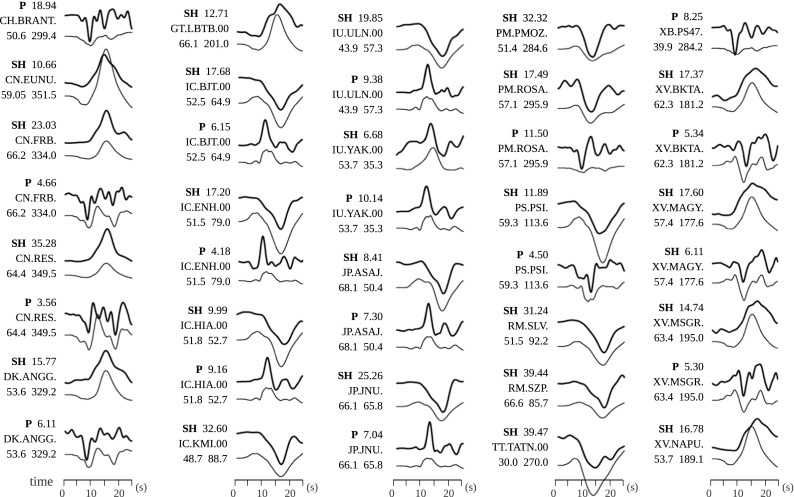
Synthetic (thin lines) and observed (thick lines) seismograms for the first event for 40 arbitrarily selected waveforms out of the 147 which were used for the final inversion. The phase of the waveform and its maximum amplitude, station code, epicentral distance, and azimuth of each station are written at the left side of the waveforms

The observed surface rupture is valuable information which makes it possible to compare the inverted slip distribution with a concrete field observation. Initially, we inverted using the values of strike, dip, rake, and focal depth reported by global-CMT (listed in Table 1). We then iteratively changed these parameters to find a good fit not only to the waveforms but also between the surface rupture implied by our inversion and the field data. Our preferred model obtained using both P- and SH-phases is shown in Fig. 2b. The best match was obtained when we used a strike of 88° and a dip of 90°, a rupture velocity of 2.8 km/s (80% of the S-wave velocity at the source) and a hypocenter depth of 12 km. These parameters resulted in a misfit of value 0.29, scalar moment of 5.49e+18 Nm, and rake value of − 170° (listed in Table 3). Our 12-km hypocenter depth is consistent with the USGS solution (11 km), with the solution obtained by simulation of strong ground motion for the first event (12 km) (Mahood et al. 2014), and is close to the relocated focal depth of 14 km suggested by Ghods et al. (2015). Figure 3 presents 40 arbitrarily selected waveforms and corresponding synthetic seismograms used to model the slip distribution of the first mainshock.

**Table 3:** Fault plane parameters for the first mainshock as suggested by waveform inversion. The hypocenter location (latitude and longitude) is from Ghods et al. (2015)

Strike/dip/ rake (°)	Hypocenter depth (km)	Hypocenter latitude (degree)	Hypocenter longitude (degree)	Rupture velocity (km/s)	*Mw*	Scalar moment (N m)	Misfit
88/90/− 168	12	38.40	46.84	2.8	6.42	5.36e+18	0.29

The slip pattern resolved for the first event (Fig. 2b) can be considered as two main zones or asperities, which we will refer to as the main patch and the side patch. The main patch includes the largest slip values, produces the surface slip and extends down to a depth of 12–14 km, with a lateral extension of 25 km along strike. The maximum slip takes place at around 4 km depth, comparable to the centroid depth of 6 ± 1 km found by Donner et al. (2015). The side patch appears at the west corner of the fault plane and is smaller and deeper than the main patch. It extends from 12 to 18 km in depth and about 10 km along strike. The observed surface rupture is marked on the topography profile along the fault plane in Fig. 2b. The model’s surface-slip matches the field data quite well, although the surface rupture implied by the model extends a bit farther to the east. In Fig. 2b, the aftershock activity during the first 3 months after the two main events is projected onto the slip map of the first event. Of approximately 230 reported aftershocks with local magnitudes larger than 3.0, we present only the 166 aftershocks which were relocated by Ghods et al. (2015).

The second mainshock occurred 11 min after the first and at approximately 6-km epicentral distance (Ghods et al. 2015). The short time difference means significant contamination between the body-waves of the second event with the surface waves and coda signals of the first, which limits the possibility of teleseismic body-wave inversion for the second event. Inversion results for the second event appear reasonable, but the interference effects make assessment of the reliability of the results difficult. In addition, there is no independent information available for this event, such as the trace of surface rupture or any distinguishable pattern of aftershock activity, to help assess model reliability. Therefore, we do not present the slip inversion analysis for the second mainshock.

## Sensitivity analysis

**Fig. 4:**
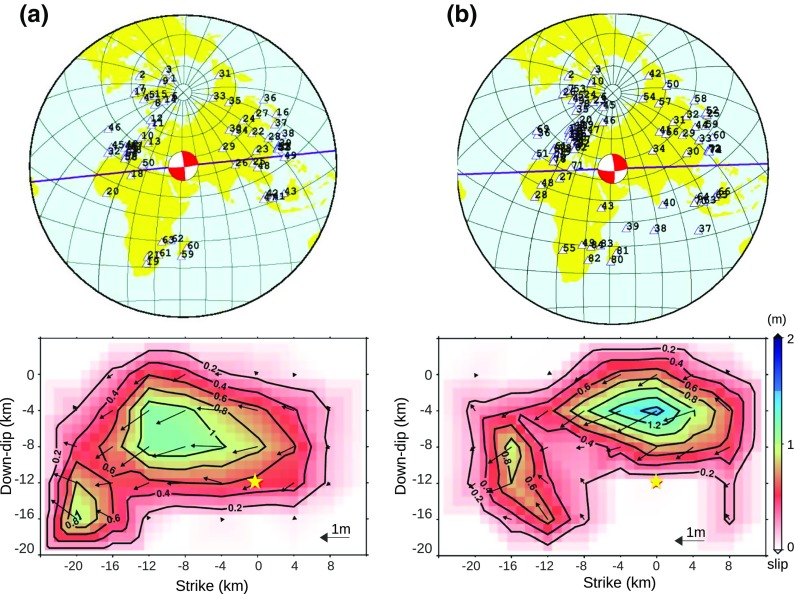
Station distribution and the estimated slip pattern of the first mainshock using **a** only SH data and **b** only P data. Markings and colors as in Fig. 2b

To assess the reliability of the analysis we analyzed the P-wave and SH-wave information, separately as well as jointly. Using identical selection criteria as for the joint inversion, 25-s data segments of 63 SH-waveforms and 84 P-waveforms for the first main shock were selected after filtering 0.015–0.5 Hz. For these time segments, the SH waveforms are simpler in character than the corresponding P. Various possible sources of noise bias can be expected to be different for the P and S analyses, and therefore, to help assess the reliability of our results, we compare the main features in the models produced using the SH and P data separately (shown in Fig. 4). We see that all inverted models are similar in that they show two distinct patches, one containing the largest slip values (the main patch) lying in the center of the fault-plane and the other with smaller spatial extent and slip values, rupturing the deeper parts of the fault plane to the west. We used the same values of the predefined parameters as for the joint P-SH inversion described above (Fig. 2b). Again, we tried station-weighting to compensate for the uneven station distribution, and again observed negligible changes in the results with and without station-weighting. Even though the SH model (Fig. 4a) looks very similar to the P-SH model (Fig. 2b), there are a few possibly significant differences such as that the magnitude of the surface slip is about 0.2 m smaller, and the side patch is stronger in the SH-only model. For the P-only model (Fig. 4b), the side patch looks somehow different from the P-SH model as it extends near-vertically toward the surface and appears closer to the center of the fault-plane. The main patch also seems to be shifted to the east in the P-only model, compared to both the P-SH and SH-only models. The maximum slip is 1.4 m in the P-only model which is 0.2 m greater than the maximum slip in the P-SH model; however, the spatial extension of the main patch is smaller in the P-only model. The surface rupture implied by the P-only model is longer than that observed in field and in the P-SH model and is also moved to the east. The misfit value obtained for the P-only model is 0.6, which is almost double that for SH-only model (0.28) and P_SH model (0.29). Hence, the P-only model can be considered less reliable than the others.

**Fig. 5:**
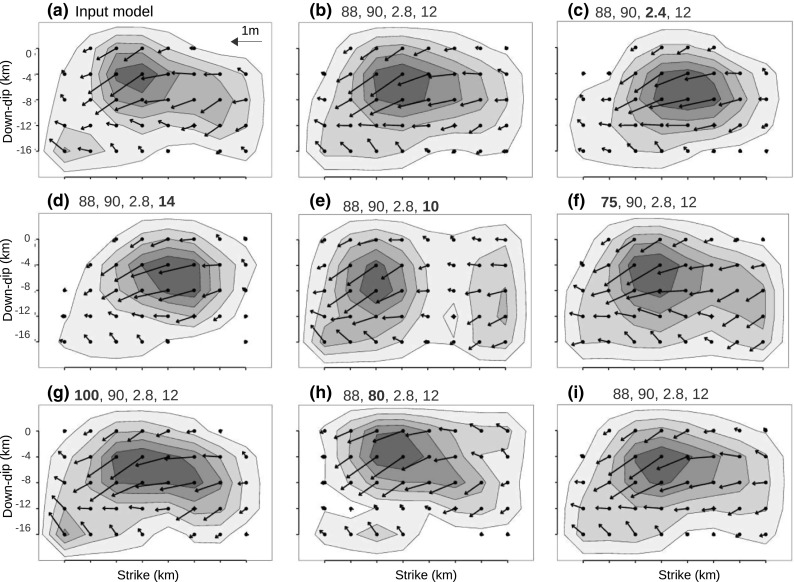
Sensitivity of the joint P-SH inversion to selected parameters. The numbers above each plot are strike, dip, rupture velocity, and hypocenter depth, respectively. These values are listed in Table 4. **a** The reference model. The modified parameter of each model is marked with a bold number. In model **i**, white noise has been added to the input waveforms. Detailed explanations about the panels are given in the text

**Fig. 6:**
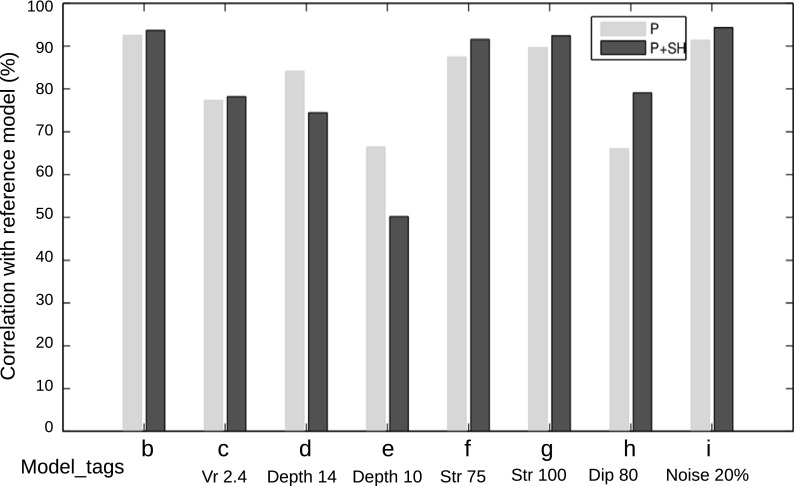
Correlation diagram, demonstrating the correlation of the slip between the reference model (Fig. 5a) and the others (Fig. 5b–i). Dark bars are the correlation values for joint inversion of P and SH waves. Light bars are correlation values for inversions using only P-waves, all other parameters identical to the dark bars

To assess the sensitivity of the inversion results to the chosen input parameters, we conducted many tests by generating synthetic data using the slip model shown in Fig. 2b and then inverting with different values of fault strike, dip, rake, rupture velocity, and hypocentral depth. We used the same station distribution as for the real data, and initially (for comparison) the same source parameters as used to generate the synthetic data. The input parameters were then changed individually to assess their influence on the inverted slip distribution. These tests are summarized in Figs. 5 and 6 and Table 4. Figure 5a shows the reference model which was used to generate the synthetics. Figure 5b is the output of the P-SH inversion with all the parameters equal to the reference model. To compare similarity between the slip models, we used the correlation coefficient between two models A and B defined as $\frac{\sum \left({A}_i-{A}_{mean}\right)\left({B}_i-{B}_{mean}\right)}{\sqrt{\left(\sum {\left({A}_i-{A}_{mean}\right)}^2\right)\left(\sum {\left({B}_i-{B}_{mean}\right)}^2\right)}}$ where *A*_*i*_ and *B*_*i*_ are slip values of the *i*th grid point, and *A*_*mean*_ and *B*_*mean*_are mean slip values over each slip model. Even though the slip variations in the inverted model (Fig. 5b) are smoother than the reference model, there is 94% correlation between models shown in Fig. 5a, b which means they are almost identical. This indicates that the station coverage, methodology, etc. are adequate to reconstruct the input source model well. However, the smoothness of the slip variations in the inverted models causes the side patch and main patch to coalesce. We also note with interest that the surface slip in the inverted model (Fig. 5b) extends slightly farther to the east than in the input model (Fig. 5a), indicating that the inversion puts more slip to the east than what is really there, consistent with the comparison of the field-observed slip and the results of our earlier inversion (Fig. 2b). In Fig. 5c, we present inversion results produced with rupture velocity decreased to 2.4 km/s (0.4 km/s lower than in the reference model) and in Fig. 5d with hypocentral depth increased to 14 km (2 km deeper than the reference model). In both cases, correlation with the input reference model was about 75%. In contrast to increasing the hypocenter depth, decreasing it by the same amount (2 km), significantly changed the inverted model and lowered the correlation value to 50% (Fig. 5e). Furthermore, we found that an incorrect choice of dip has more influence on the results than a similar error in strike. For example, changing the strike value by 10° (Fig. 5f, g), resulted a correlation of 92%, whereas changing the dip value (Fig. 5h) by the same amount decreased the correlation value to 79%. In general, we can say that the inversion is more sensitive to incorrect choice of the hypocentral depth and the dip value than to incorrect values of strike and rupture velocity.

**Table 4:** Inversion parameters used for synthetic tests

Model	b	c	d	e	f	g	h	i (20% noise)
Strike (°)	88	88	88	88	*75*	*100*	90	90
Dip (°)	90	90	90	90	90	90	*80*	90
Rupture velocity (km/s)	2.8	*2.4*	2.8	2.8	2.8	2.8	2.8	2.8
Depth (km)	12	12	*14*	*10*	12	12	12	12
Misfit	0.002	0.003	0.015	0.005	0.10	0.08	0.045	0.24
Correlation (%)	94	78	75	50	91	80	79	94

Italic numbers mark the modified parameter

We also assessed the sensitivity of the inverted slip model to noise in the data by inverting synthetic data with added random (Gaussian) white noise. We tried adding noise with different signal to noise ratios. After adding noise to the synthetic waveforms the same band-pass filter as used for the real data was applied before inversion. Here, we present results with 20% added noise (signal to noise ratio is 5) which produced a model that correlated to 94% with the reference model (Fig. 5i), but has a misfit value of 0.24. This means that the inversion can properly handle the random noise in the input data with minor effects on the resulted slip map but noticeable increase in the misfit value. The misfit value of the real inversion, 0.29, is comparable to the misfit value of the model shown in Fig. 5i.

The same sensitivity analysis was implemented for the inversion with only P-data to compare the stability of the inversion results with the P-SH data. The slip results are presented in online resource 1 (Fig. S2), but the correlation values are included beside the P-SH models in Fig. 6 (light bars). As illustrated by Fig. 6, except for the changes of hypocentral depth, the correlation values are higher for P-SH inversion than for the corresponding P-only inversion. Furthermore, the surface rupture also is more sensitive to the change of parameters in P-only inversions (online resource 1, Fig. S2) than for the P-SH inversions. These results confirm that the P-SH inversions are more stable than the P-only inversions, thus supporting our choice of the P-SH model as the most reliable one. We also conducted tests for the SH-only inversions, but as the effects are rather similar to the P-SH inversions, so we do not present the results here.

## Coulomb stress results

**Fig. 7:**
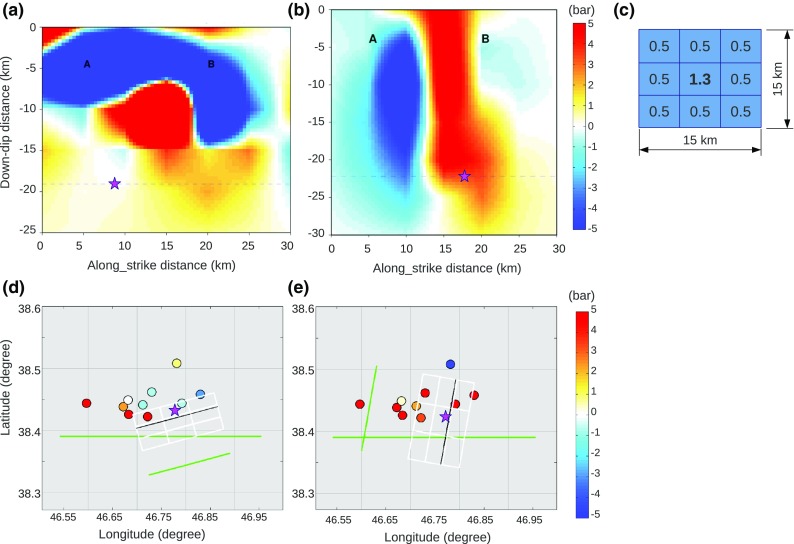
Coulomb stress changes caused by the first mainshock on the two nodal planes of the second mainshock, **a** nodal plane 2a, **b** nodal plane 2b. **c** Assumed slip distribution for the second event. The number in each cell is the net slip value, and the slip direction is according to the global CMT rake. The Coulomb stress changes on nodal planes of the largest aftershocks, considering **d** the slip distribution from event one and nodal plane 2a from event two as the source faults and **e** event one and nodal plane 2b as the source faults. Star is the second mainshock; circles are the ten largest aftershocks

Using the slip inversion results for the first event (P-SH model, Fig. 2b) as the source and the second event nodal planes as the receivers, we calculated the Coulomb failure stress (ΔCFS) on the two nodal planes, faults 2a and 2b, of the second event. The orientations of the nodal planes for the second event are based on the global-CMT solution. Sections of Coulomb stress along these two planes are shown in Fig. 7. Shear failure is encouraged where the Coulomb stress change is positive (red) and is discouraged where it is negative (blue). The estimated initial rupture location of the second event at depth 17 km, obtained from Ghods et al. (2015), is shown by the pink star. With the E-W nodal plane of the second event (fault 2a), the Coulomb stress changes are almost neutral around the hypocenter (Fig. 7a), while they are clearly positive when the hypocenter is on the 2b fault-plane (Fig. 7b). The hypocenter on the fault 2b plane would remain in the positively induced area for all depth estimates from surface down to about 35 km and for ± 3-km variations along the strike. However, we do not consider that these results alone are sufficient to prefer plane 2b to 2a, because shear failure is not discouraged on the 2a plane.

We can obtain an additional indication on which plane is the fault plane, from the combined Coulomb stressing effect of the two main events on the location of the aftershocks. Focal mechanism information is available from Donner et al. (2015) for the ten largest aftershocks (*Mw* > 4.2). All but one of these occurred within 5 days of the mainshocks, with the one 3 months later (2012.11.07) being the largest aftershock of the sequence (*Mw* 5.6). In order to perform Coulomb stress calculations for the aftershocks, we need a model of the slip distribution in the second events as well as the first. Since we do not have a slip pattern available for the second event, we assume one using the focal mechanism solution and scalar moment info from global-CMT, the calibrated hypocenter location from Ghods et al. (2015), and a hypothetical slip area of 15 km by 15 km, consistent with extension of the rupture area found in the GPS study (Yadav et al. 2016). We gridded the fault plane into three by three cells with constant slip value in each cell and assumed that the highest slip occurs in the central cell, which is just above the hypocenter (Fig. 7c). We chose this because of the shallower centroid depth (12–14 km, Donner et al. 2015) relative to the hypocenter depth (17 km, Ghods et al. 2015), and also because the main slip happens at above 15-km depth in the slip model of the second event presented by Yadav et al. (2016) using GPS measurements. Having a slip model for the first event and two alternative models (2a and 2b) for the two nodal planes of the second event, we can calculate the Coulomb failure stress change at the reported locations and fault orientations of the aftershocks in their rake directions. Toda et al. (2011a) used a similar approach for the 2011 *Mw* 9.0 Tohoku earthquake and showed a reasonable gain in positively stressed aftershock nodal planes affected by the mainshock. We perform the calculations for both nodal planes of the Varzeghan aftershocks and the nodal plane with the largest Coulomb stress changes is chosen as the fault plane and shown in the results (Fig. 7d, e). We find that 9 out of 10 of the aftershocks have positive Coulomb stress changes when nodal plane 2b of the second event is used, but only half of them are positive if 2a is used instead. To test the robustness of the result, we repeated the calculations assuming uniform slip distribution on the nodal planes of the second event, which means a net slip of 0.6 m for all cells in Fig. 7c. The number of aftershocks with increased/decreased Coulomb failure stress changes remained the same for both nodal plane 2a and 2b. These results indicate that from a Coulomb stress analysis point of view it is likely the N-S nodal plane is the fault plane that ruptured in the second event.

## Discussion

One of the initial questions that arises in earthquake studies is identifying the causative fault of the earthquake. In the Varzeghan doublet, finding the slip plane of the first event is straightforward due to the clear fault trace at the surface, but for the second event is a challenging question. For the first mainshock, teleseismic body-wave inversion provided the slip distribution on the fault plane. A joint inversion of P- and SH-wave data resulted in more robust results than using only P-waves, but also more detail than using only SH-waves. Despite the intrinsic non-uniqueness of inversion for slip, the waveform inversion provided reasonably stable results for the first event, results which are consistent with the observed surface rupture and the aftershock distribution, and also comparable to other studies in terms of the hypocenter depth (Ghods et al. 2015; Mahood et al. 2014), centroid depth (Donner et al. 2015) and the resolved slip pattern (Yadav et al. 2016). The deduced slip pattern (Fig. 2b) shows two distinct slip patches: the main patch and the side patch. The side patch ruptures a deeper part of the fault with smaller slip values than the main patch. The sensitivity analysis suggests that the side patch could be even stronger than what appears in the slip map, since it has been smoothed out in the inversion process (compare Fig. 5a, b). We tested the idea by increasing the length of the fault plane along the strike to the west and observed a stronger (wider extent and higher slip values) side patch which also resulted in an increased moment magnitude, closer to the value reported by global-CMT. Furthermore, applying different station-weighting schemes to bias the azimuthal coverage (online resource 1, Fig. S1) results in an enhanced side patch. However, the deduced slip pattern is comparable to the one obtained from near-field GPS measurements (Yadav et al. 2016) where the main slip is between the surface down to 15-km depth with highest slip values concentrated between surface down to about 10 km. The GPS-derived slips also indicate a small slip patch at the western-bottom end of the fault (Fig. 3a in Yadav et al. 2016), comparable with our side patch. We suspect that the side patch could be coseismic slip on secondary faults since its predominant slip direction is different (upward) to that on the main patch (horizontal).

By definition, “a double-event” is two successive earthquakes with comparable magnitudes in which the second event is in some sense a consequence of the first, and they are close in space and time (Davidson 1905). The 11-min time delay, 6 km horizontal separation of epicenters and intersecting fault planes puts the Ahar-Varzeghan mainshocks in the doublet category. The calculations of Coulomb stress changes for the doublet suggests that the N-S nodal plane of the second event is the fault that ruptured. The choice of the N-S nodal plane is also supported by the results of surface wave inversion and first motion body-wave polarities (Donner et al. 2015). However, it is not in agreement with the Ghods et al. (2015) study, which deduced that only the E-W fault plane of the second event is compatible with the regional stress state obtained from inversion of geological and seismologically determined fault kinematics. Investigating this apparent misfit, we calculated slip directions on the two nodal planes using the stress field (both the geologically and the seismologically determined) from Ghods et al. (2015). The results show similar fits for both nodal planes, although the slip direction predicted on the N-S nodal plane actually fits the observed rake slightly better than that on the E-W plane. These results of course depend to some degree on the assumed strike, dip and coefficient of friction, but the main conclusion of similar fits on both nodal planes remains.

**Fig. 8:**
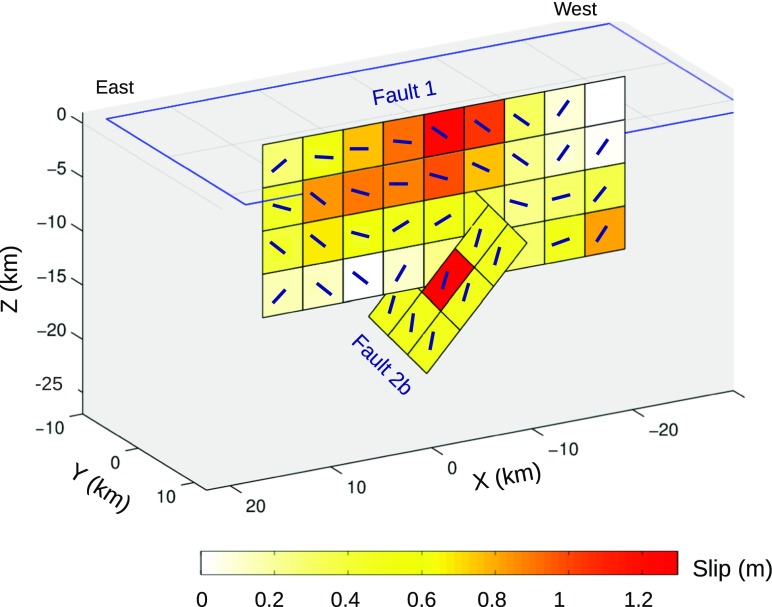
3D slip maps of the doublet illustrating the fault plane 1 from event one and nodal plane 2b from event two. Color scheme shows the amount of net slip on each fault segment. Lines are the slip direction in each cell

Figure 8 shows 3D slip maps of the first (fault plane 1) and the second (fault plane 2b) mainshocks. The slip distribution of the first fault is the one estimated using body-wave waveform inversion and for the second faults it is the one used in our Coulomb modeling, described above. The two faults apparently intersect at the depth where the slip values of the first event are not high. The slip model for the first event also shows no rupture above the side patch. This zone of no slip coincides with a north-south extending cloud of aftershocks covering the western end of the fault plane (Fig. 1c). This aftershock sequence is associated with the *Mw* 5.6 event that occurred just above the side patch, 86 days after the doublet and was followed by more than 150 aftershocks (Ml > 2.0) in just a week after.

In general, aftershock activity is prone to occur in areas with high stress concentrations produced by the mainshock (Mendoza and Hartzell 1988). If we attribute all the relocated aftershocks to the first mainshock and project the seismicity onto the deduced slip pattern (Fig. 2b), it appears that the aftershocks tended to not occur in the areas of high slip but are mainly located in areas with lower slip. Such patterns of aftershock distribution have been observed several times, e.g., for the Loma-Prieta earthquake in 1989 *Mw* 7.0 (Beroza 1991) and Morgan-Hill earthquake in 1984 *Mw* 6.2 (Bakun et al. 1986). The aftershocks of the Ahar-Varzeghan doublet earthquakes show a diversity of focal mechanism solutions (Donner et al. 2015; Ghods et al. 2015) varying from pure strike-slip to oblique-thrust and thrust events. This, together with the resolved side patch on the first fault-plane that shows different slip direction compared to the main patch, and also the fact that we have a doublet with dissimilar faulting mechanisms, all suggest a complex deformation style where the coseismic activity is not limited to the primary fault planes and is taking place on subsidiary faults as well. Other studies regarding the Varzeghan doublet (Donner et al. 2015; Ghods et al. 2015) have also suggested a very complex style of deformation for the area.

We can also consider the implications for hazard in the area by considering the Coulomb stress changes that the double events have induced on the neighboring faults. The most important of these is the North Tabriz Fault which, based on recent GPS measurements (Djamour et al. 2011) and elastic dislocation modeling of InSAR data (Karimzadeh et al. 2013), has an average slip accumulation rate of 7–8 mm/year. This average rate suggests a recurrence time of 250–300 years if all of the accumulated shear stress is released by magnitude 7–7.4 earthquakes. This is consistent with historical seismicity data. The last two major earthquakes on the North Tabriz Fault were in 1721 (magnitude 7.3) and 1780 (magnitude 7.4), which ruptured the southern and central parts the fault, respectively. This gives an elapse time of 293 and 234 years, implying that there could be a high risk of a significant earthquake in the coming decades. If so, any stress changes in the vicinity of the North Tabriz Fault could have important implications for the risk of failure and should be studied carefully. The double-event occurred approximately 50 km northwest of the city of Tabriz. According to field studies (Karakhanian et al. 2004), the Tabriz segment of the NTF has had predominant right-lateral strike-slip motion on near vertical faults. To calculate the induced Coulomb stress changes on the Tabriz segment of the NTF, the double event was defined as the source and vertical pure strike-slip planes as receiver faults. We tested three source fault cases as (a) source faults 1 and 2a, (b) source faults 1 and 2b, and (c) only fault 1 acting as the source. In all these cases, the induced stresses on the North Tabriz Fault are minor, primarily because of the relatively large distance between the North Tabriz Fault and the double-event (50 km). According to the Coulomb estimates the resulting stress changes are negative and below −0.03 MPa, implying that this part of the NTF has not been moved close to the failure by the doublet.

## Conclusions

On August 11, 2012, an earthquake doublet occurred in northwestern Iran, causing more than 3000 casualties in the Varzeghan city which was devoid of significant seismic activity. The first mainshock, *Mw* 6.5, is a strike-slip earthquake and can be associated with an E-W trending distinct surface rupture. The second mainshock, *Mw* 6.4, is an oblique thrust event that occurred 11 min after the first with 6-km epicentral separation. For this event, identification of the fault plane is difficult due to the absence of a surface fault plane or a clear aftershock trend. In this study, we tried to estimate the slip distribution for the first event and find the fault plane of the second event.

Teleseismic body-waves of 147 P- and SH-waveforms, obtained from IRIS at teleseismic distances (30°–70°), were inverted to find the slip distribution pattern for the first mainshock. In order to address the non-uniqueness of the results, we used the surface rupture to constrain the estimated slip model. The inversion results revealed two slip patches with different predominant slip directions. The different slip directions may imply either a complex stress situation prior to the events or significant lateral variations in shear strength along the fault.

To find the fault plane of the second mainshock we considered static triggering via applying Coulomb failure criteria. We considered the coulomb stress changes from the first event on nodal planes of the second. This analysis showed that the N-S nodal plane is more unstable than the E-S plane. We then estimated the Coulomb stress changes following the doublet, on the nodal planes of the largest aftershocks. To assess the combined Coulomb stress field, we assumed a simple slip pattern on two candidate nodal planes of the second event. Nine out of ten of the aftershocks were brought closer to failure when the N-S nodal plane of the second event was used as the source fault. Only five of the aftershocks showed positive stress changes with the E-W plane, suggesting the N-S plane is the correct fault plane for the second event. The effect of the doublet on the stability of the North Tabriz fault is estimated by calculating Coulomb stress changes due to the double event on vertical strike-slip receiver faults. We find that the North Tabriz Fault has not been brought closer to failure by the doublet.

## Electronic supplementary material

ESM 1 (PDF 395 kb).
