# Supplementary material for: Fault slip and identification of the second fault plane in the Varzeghan earthquake doublet
Source: J Seismol. 2018 Feb 22;22(4):815–31. doi: 10.1007/s10950-018-9734-0 (PMC6015626; doi:10.1007/s10950-018-9734-0)
Supplement: Supplementary file 1 — (PDF 395 kb). [file 10950_2018_9734_MOESM1_ESM.pdf]

Electronic supplementary material for

Fault slip and identification of the second fault plane in the Varzeghan earthquake doublet

Samar Amini[1], Roland Roberts[1], Mohammad Raeesi[2], Z. Hossein Shomali[1], Bjorn Lund[1], Zoya Zarifi[3]

[1]Department of Earth Sciences, Uppsala University, Uppsala, Sweden

[2] Independent Researcher, PhD in Seismology

[3]Department of Earth Sciences, University of Western Ontario, London, Ontario, Canada

Corresponding author: S. Amini, Department of Earth Sciences, Uppsala University, Villavagen 16, 75236 Uppsala, Sweden,Telephone +46 18 4712381, Fax +46 18 501110

Introduction

Two figures have been presented in this document to support the discussions in the main text. The first figure demonstrates the effect of a few weighting schemes applied for the slip inversion. The second figure shows sensitivity of the P-only slip model to the input parameters of the inversion.

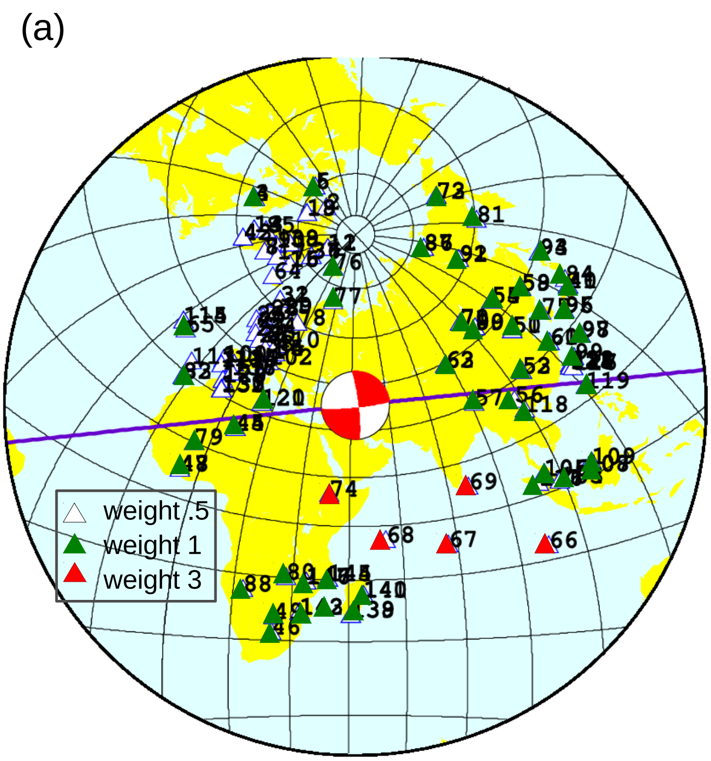

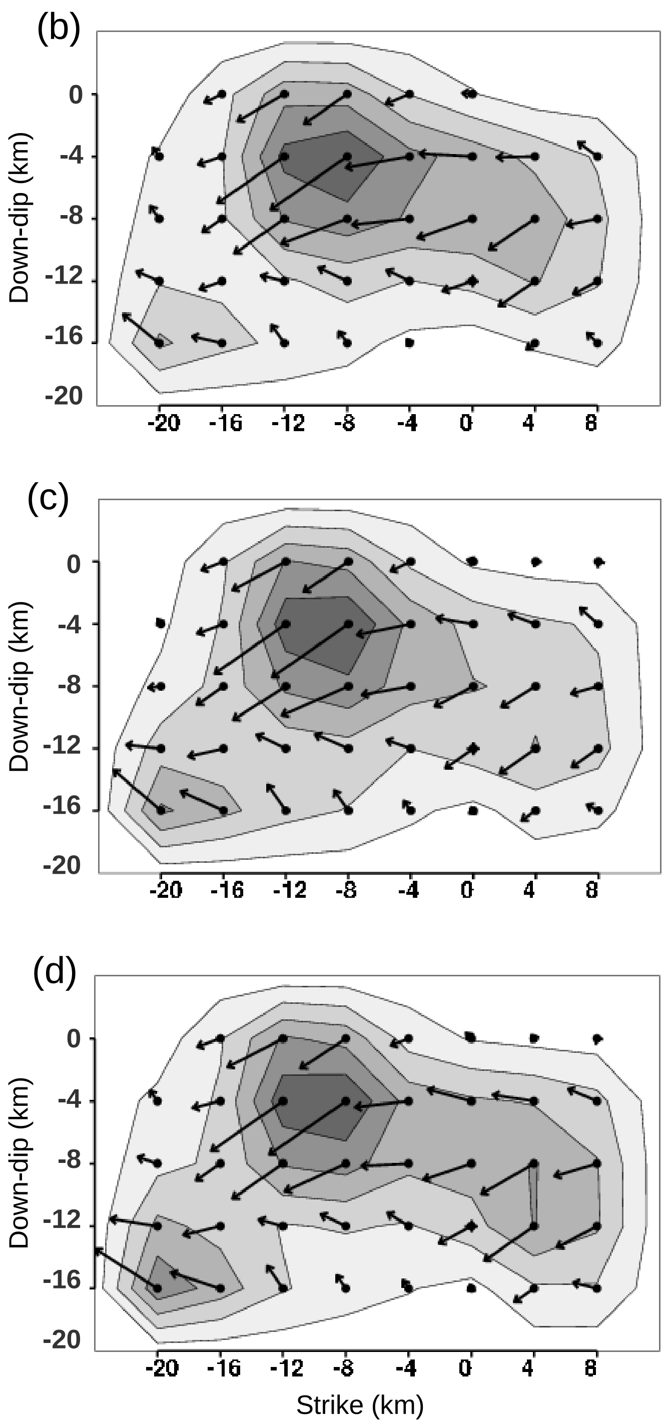

Fig S1. Joint inversion of P and SH data for slip distribution of the first main-shock. (a) station distribution used for the inversion. Color assigned to each station marks its weighting value. (b) Slip distribution map obtained when all waveforms had the same weighting. (c) slip distribution after applying the weighting scheme demonstrated in (a). (d) slip distribution after applying weighting 0.5 for all the stations marked with white triangle in (a) and weighting one for the others.

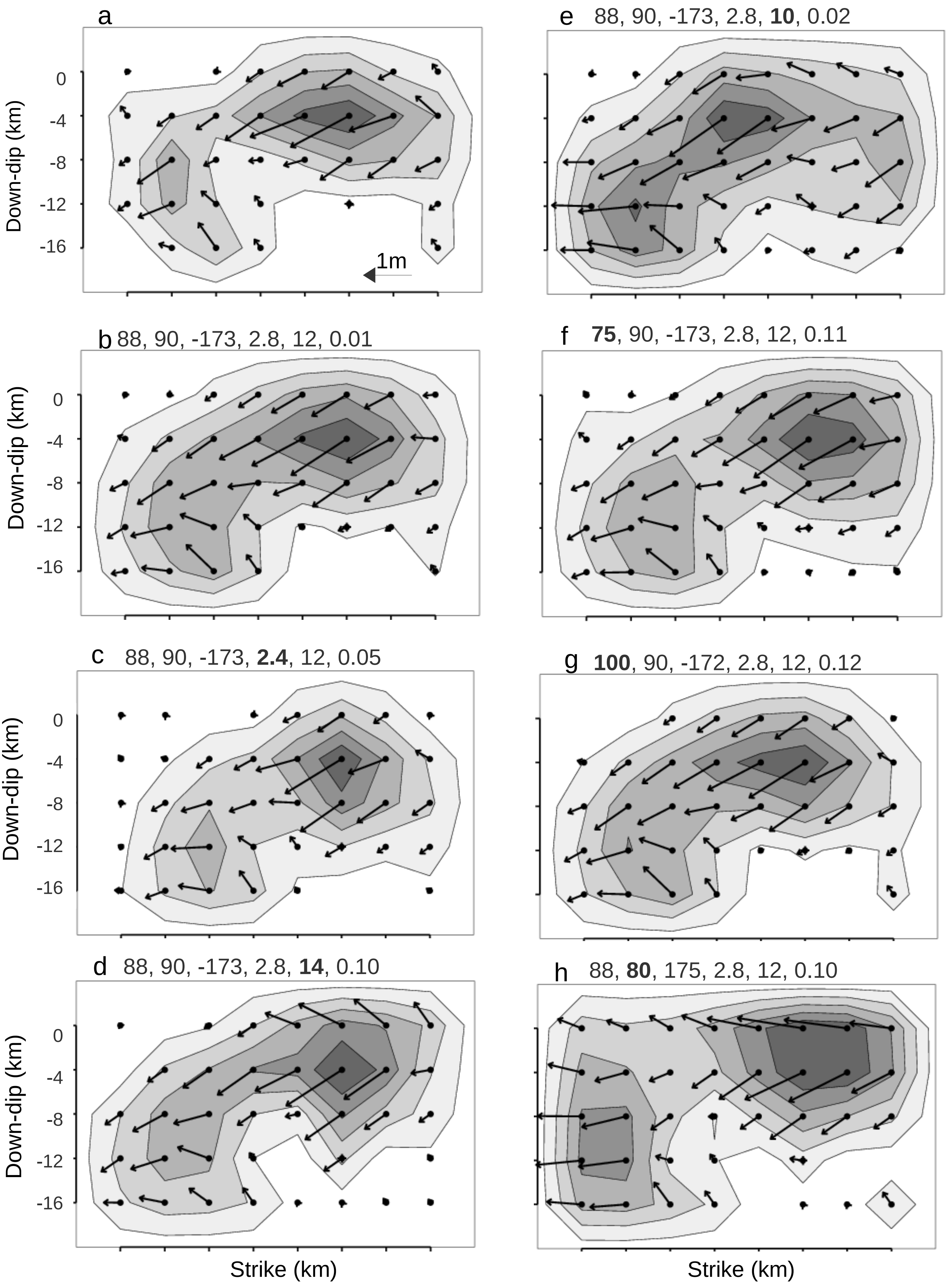

Fig S2. sensitivity of the P_only inversion results to the preselected parameters. The numbers above each plot are strike, dip, rupture velocity, hypocenter_depth, and the resulted misfit value, respectively. (a) is the reference model. The modified parameter of each model is marked as bold number.
